# Totally Tubeless Outpatient Percutaneous Nephrolithotomy: Initial Case Report

**DOI:** 10.1155/2009/295825

**Published:** 2009-05-24

**Authors:** Darren Beiko, Meghana Samant, Thomas B. McGregor

**Affiliations:** Department of Urology, Kingston General Hospital, Queen's University, 76 Stuart Street, Kingston, ON, Canada K7L 2V7

## Abstract

We report the first case of totally tubeless outpatient percutaneous nephrolithotomy (PCNL). Our patient was discharged home safely less than 4 hours following uncomplicated PCNL with no nephrostomy tube, ureteral stent, or urethral catheter. Follow-up the next day in clinic confirmed that the procedure was successful, as the patient was clinically well and stone free. To our knowledge, this is the first case report of totally tubeless (no nephrostomy, no ureteral stent) PCNL performed on a truly outpatient basis.

## 1. Introduction

Most endourologic procedures can be done on an outpatient basis, yet patients requiring PCNL are routinely admitted to hospital postoperatively. With the advent of tubeless PCNL [3–6] and more recently, totally tubeless PCNL [7–10], postoperative length of stay following PCNL has decreased. Singh et al. have reported “ambulatory” PCNL [1], however when one looks closely at their report, each patient was discharged home the following day. We recently reported a successful case series of tubeless outpatient PCNL in three cases [2], all of which involved ureteral stent placement. This case report represents the first known case of totally tubeless (no percutaneous nephrostomy tube or ureteral stent) PCNL performed on an outpatient basis.

## 2. Case Report

**Figure 1:**
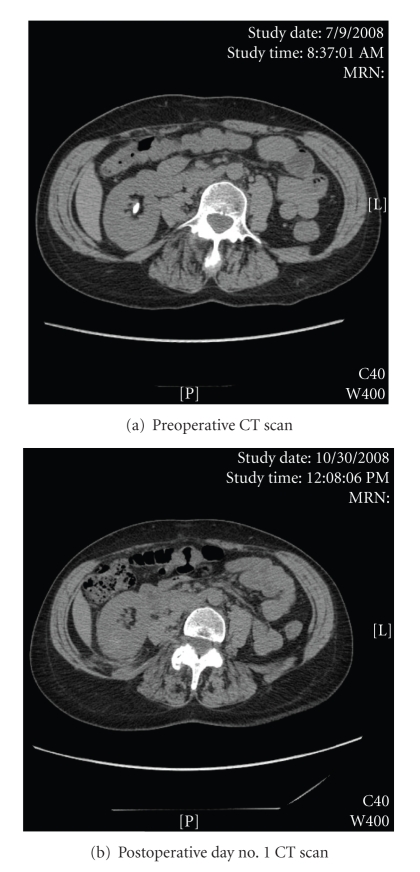


A generally healthy 58-year-old woman presented with a 3-month history of intermittent right flank pain and several episodes of intermittent gross hematuria. On physical examination, she appeared well and her stated age. Pre-operative blood pressure was 100/72 mmHg, and pre-operative heart rate was 78 beats per minute. Urinalysis revealed microhematuria, and urine culture was negative. Pre-operative bloodwork revealed a hemoglobin of 147 g/L and a creatinine of 56 *μ*mol/L. KUB and CT scan (Figure 1(a)) revealed an 11 × 5 mm right lower pole calculus with no hydronephrosis. At our institution, all patients with lower pole stones greater than 1 cm in maximum diameter are offered shock wave lithotripsy, ureterorenoscopic laser lithotripsy, and PCNL. The patient stated very clearly that she was not interested in passing stone fragments, and she wanted the most definitive procedure that would best ensure complete removal of her stone in a single setting, a PCNL. Informed consent was obtained for PCNL, and she was treated with oral levofloxacin 500 mg daily for seven days prior to the operative procedure.

Intraoperatively, intravenous Ampicillin 1 g and Gentamicin 80 mg were administered. The procedure was performed in the prone position. Flexible cystourethroscopy was normal. Retrograde pyelogram showed the stone in a lower calyx. Percutaneous access was achieved at the tip of an interpolar calyx because its infundibulum was wide and there was a wide angle between it and the lower pole calyx harbouring the stone. The tract was dilated using a 30 French balloon dilator. Using a 24 French rigid nephroscope, the stone was readily identified and removed intact using duckbill graspers. There was negligible bleeding during the procedure and negligible bleeding on inspection of the tract at the end of the procedure. Therefore, decision was made to not leave any indwelling nephrostomy tube or ureteral stent. Total operative time, including cystoscopy and retrograde pyelogram, was 47 minutes.

During the early postoperative period, her vital signs were stable, and there was minimal pain and hematuria. The urethral catheter was removed prior to discharge, and the voided urine was pink in colour. She was discharged home 72 minutes after being discharged from the recovery room and less than four hours after leaving the operating room. At time of discharge, arrangements were made for a follow-up telephone call later that evening and postoperative assessment the following day as an outpatient. Assessment on postoperative day no. 1 included bloodwork, noncontrast CT scan and a clinic appointment with the attending urologist.

**Figure 2:**
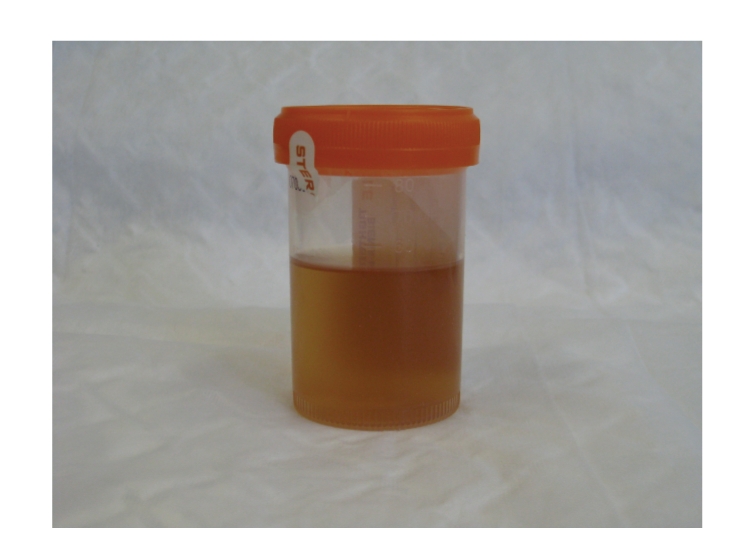
Urine sample on postoperative day no. 1.

The patient had no complaints at telephone follow-up and clinic follow-up on the first postoperative day. In the first 24 hours following hospital discharge, the patient had very little discomfort, as evidenced by the fact that she took only four acetaminophen 500 mg tablets and absolutely no narcotics. On examination, she appeared well, her vitals signs were stable, and her incision was clean and intact. A urine sample revealed minimal hematuria (Figure 2). Her hemoglobin was 127 g/L, and her creatinine was 73 *μ*mol/L. A CT scan showed a normal kidney with minimal postsurgical changes in the tract but no evidence of any significant hematoma, fluid collections, or residual stones (Figure 1(b)). Stone analysis proved it to be a calcium oxalate stone.

## 3. Discussion

Although generally safe and effective, PCNL can be associated with significant morbidity, including infection, hemorrhage, urinary obstruction, and urinary leakage. To prevent these complications, placement of nephrostomy tubes is standard practice following PCNL in most centers. Although nephrostomy tubes provide postoperative drainage of urine from the collecting system, they can cause discomfort and increase hospital stay.

In attempt to reduce morbidity from nephrostomy tubes, tubeless PCNL emerged, which initially involved using ureteral stents for renal drainage in place of nephrostomy tubes [3–6]. These initial studies showed that tubeless PCNL was safe and effective. Since that time, some groups started performing totally tubeless PCNL, questioning the need for any type of drainage following PCNL [7–10]. However, such patients were still routinely admitted postoperatively.

We recently reported our experience with outpatient tubeless PCNL in a very small series of patients [2]. These patients had placement of a ureteral stent intraoperatively at the end of their PCNL procedure in order to ensure adequate renal drainage. As a result of the success in our small case series, combined with the straightforward and uncomplicated nature of this particular patient's operation, we elected to attempt and to perform an outpatient PCNL without any nephrostomy tube or ureteral stent. All of our previously published discharge criteria [2] were met, and we arranged outpatient follow-up on postoperative day no. 1.

Our patient did not have any significant pain postoperatively, nor did she endure any minor or major complications. She experienced minimal hemorrhage as evidenced by her minimal and transient gross hematuria and her postoperative hemoglobin (Table 1). CT scan confirmed that she was stone-free.

This case report represents the first successful case of totally tubeless PCNL performed on a completely outpatient basis. The role of totally tubeless outpatient PCNL in larger stones has yet to be determined. We propose that this approach could be extended to larger stones and needs to be studied in a larger and more diverse patient population before it is widely employed as standard treatment.

## Figures and Tables

**Table 1:** Pre-operative and postoperative clinical parameters.

	Pre-operative	Postoperative
Pain	Moderate, intermittent	Very mild
Heart rate, (beats per minute)	78	72
Blood pressure (mmHg)	100/72	106/60
Hemoglobin (g/L)	147	127
Creatinine (*μ*mol/L)	56	73
CT scan	11 × 5 mm lower pole stone	Stone-free, minimal perinephric stranding along tract
